# Identification of a novel redox switch between metabolism and cardiac function using HyPer power

**DOI:** 10.1007/s00424-023-02832-w

**Published:** 2023-06-23

**Authors:** Henning Morawietz

**Affiliations:** Division of Vascular Endothelium and Microcirculation, Department of Medicine III, Faculty of Medicine and University Hospital Carl Gustav Carus, TUD Dresden University of Technology, Fetscherstr. 74, 01307 Dresden, Germany

**Keywords:** Cardiac function, Hydrogen peroxide, Isocitrate dehydrogenase, Metabolism, Redox signaling

Commentary on: Nanadikar MS, Vergel Leon AM, Guo J, van Belle GJ, Jatho A, Philip ES, Brandner AF, Böckmann RA, Shi R, Zieseniss A, Siemssen CM, Dettmer K, Brodesser S, Schmidtendorf M, Lee J, Wu H, Furdui CM, Brandenburg S, Burgoyne JR, Bogeski I, Riemer J, Chowdhury A, Rehling P, Bruegmann T, Belousov VV, Katschinski DM (2023) IDH3γ functions as a redox switch regulating mitochondrial energy metabolism and contractility in the heart. Nat Commun 14:2123.

Metabolic disorders are important risk factors for cardiovascular diseases. The underlying molecular mechanisms are not well-understood. Therefore, the search for potential links between metabolism and cardiovascular system is a hot area of research in physiology and pathophysiology. Especially the role of reactive oxygen species (ROS) in this context is controversially discussed.

ROS play a major role in the regulation of cardiovascular function and the development of cardiovascular diseases [1]. ROS include reactive free oxygen radicals like superoxide anions (O_2_^−•^) and stable non-radical oxidants like hydrogen peroxide (H_2_O_2_). Free oxygen radicals can have pathophysiological effects by reducing nitric oxide (NO) availability and increasing peroxynitrate formation and oxidative modification of different biomolecules [7]. On the other hand, H_2_O_2_ is considered as an important physiological signaling molecule due to its rather long half-life and its ability to pass membranes. Depending on its intracellular location and concentration, H_2_O_2_ could be protective or deleterious in the cardiovascular system [5]. Most studies addressing this question so far were using transgenic mouse models overexpressing or deleting the major endogenous source of H_2_O_2_ in the cardiovascular system, the NADPH oxidase Nox4. In the vasculature, Nox4 knockouts provide anti-atherosclerotic effects and endothelial protection [4, 8]. In the myocardium, the role of Nox4 is less well-defined [5]. Several studies using Nox4 knockouts showed protective effects in the heart, but upregulation of Nox4 in the myocardium can also cause cardiac remodeling via Akt-mTOR and NFκB signaling pathways [10].

The type of transgenic models is important for the analysis of ROS in the cardiovascular system [9]. Transgenic models can differ in the type of affected ROS, their intracellular or tissue-specific ROS localization, and their effect on ROS concentration. High or low ROS concentrations can mediate different, sometimes opposite effects. Low H_2_O_2_ concentrations in the nanomolar range might be protective, while high H_2_O_2_ concentration above 100 µm can induce cytotoxic effects and cell death. The sensitivity to ROS differs between cells and tissues due to their antioxidative capacity. Furthermore, the assays used to measure ROS needs to be critically discussed [2]. Taking these points into account, the development of appropriate transgenic models is crucial to test the role of H_2_O_2_ as a potential molecular switch between metabolic and cardiovascular functions in vivo (Fig. 1).

**Fig. 1:**
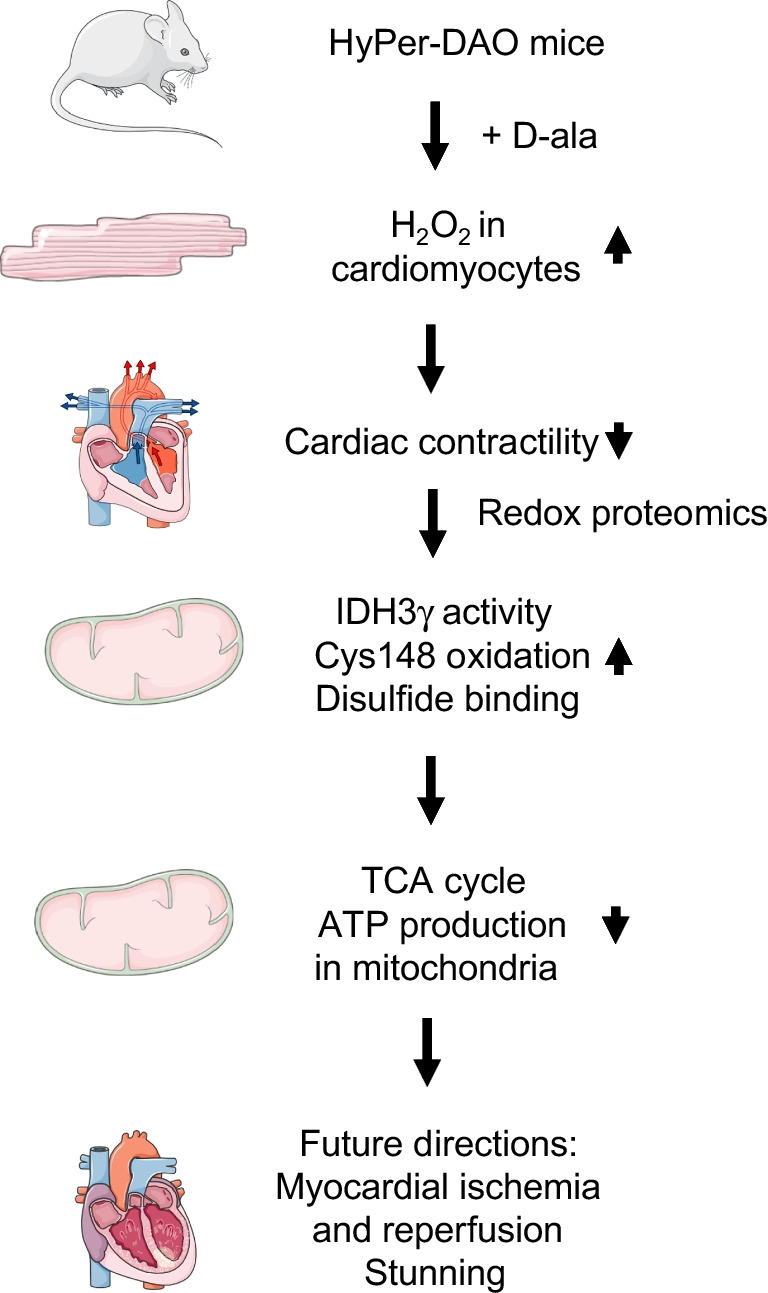
Impact of hydrogen peroxide activation in the cardiomyocytes of HyPer mice on cardiac contractility, mitochondrial isocitrate dehydrogenase 3 γ activity, ATP production, and metabolism. Parts of the figure are adapted from SMART – Servier Medical Art, Servier: https://smart.servier.com. Abbreviations: ATP, adenosine triphosphate; Cys148, Cysteine 148; D-ala, D-Alanine; DAO, D-amino acid oxidase; H_2_O_2_, hydrogen peroxide; HyPer, Hydrogen Peroxide sensor; IDH3γ, isocitrate dehydrogenase 3 γ; TCA, tricarboxylic acid

This important scientific question has been addressed by Dörthe Katschinski and her colleagues [6]. The paper has been recently published in *Nature Communications* and is highlighted as the Paper of the Month by the German Physiological Society. In this excellent study, Nanadikar et al. developed a novel chemogenetic transgenic mouse model of inducible overexpression of the hydrogen peroxide sensor HyPer using D-amino acid oxidase (DAO) (HyPer-DAO mice) in cardiomyocytes. The primarily nuclear localization of HyPer in this model resulted in a better fine tuning of intracellular H_2_O_2_ formation in comparison to classical overexpression or knockout of endogenous H_2_O_2_ sources like Nox4. The HyPer probe was reversibly inducible in response to the DAO-specific substrate D-Alanine (D-ala). Using this model, increased endogenous H_2_O_2_ formation in cardiomyocytes was leading to impaired cardiac contractility. The authors identified novel redox-sensitive target proteins by proteomics. Hydrogen peroxide could reversibly oxidize protein cysteine thiols (–SH) to sulfenic acid (–SOH) finally resulting in disulfide-bridge formation. Such a reversible oxidation of a protein cysteine thiol by H_2_O_2_ is considered as a redox switch. Most of the redox-sensitive proteins identified in this screen had cysteine residues. The authors focused on proteins involved in mitochondrial metabolism and discovered the γ-subunit of the TCA cycle enzyme isocitrate dehydrogenase (IDH) 3 as a novel redox switch linking metabolism and cardiac function. HyPer-DAO overexpressing HEK cells exhibited a reversible redox modification and activity of IDH3 after activation of DAO in vitro. Endogenously produced H_2_O_2_ impaired ATP generation in the mitochondria. In a series of elegant experiments using microsecond molecular dynamics simulations and cysteine-gene-edited cells, they could prove that IDH3γ cysteine (Cys) 148 and 284 are critically involved in the H_2_O_2_-dependent regulation of IDH3 activity. Finally, they provide evidence that the redox modification of IDH3γ Cys148 and Cys284 is responsible for ATP production in the mitochondria. In summary, these data shed light into a novel mechanism how mitochondrial metabolism could be linked via redox-sensitive mechanisms to cardiac function.

What are the implications of these exciting findings for cardiovascular physiology and pathophysiology? First, redox-sensitive modifications of these and other proteins or cysteine residues could provide novel regulatory mechanisms to fine tune the cross-talk between metabolism and cardiac function. Second, this novel transgenic mouse model might be used to study the role of H_2_O_2_ in important processes like myocardial ischemia and reperfusion. Especially interesting would be a detailed molecular analysis of the most probably redox-sensitive mechanisms of stunning [3].

In conclusion, the use of HyPer power might provide stunning novel views into redox-sensitive processes of cardiovascular physiology and pathophysiology.

## Data Availability

Not applicable.
